# Turnbull–Cutait technique without ileostomy after total mesorectal excision is associated with acceptably low early post‐operative morbidity

**DOI:** 10.1111/ans.16412

**Published:** 2020-10-30

**Authors:** Osman Serhat Guner, Latif Volkan Tumay

**Affiliations:** ^1^ Department of Surgery Acibadem Bodrum Hospital Bodrum Turkey; ^2^ Operating Room Services Acibadem University, Vocational School of Health Sciences Istanbul Turkey; ^3^ Department of Surgery Acibadem Bursa Hospital Bursa Turkey

**Keywords:** coloanal anastomosis, early post‐operative morbidity, ileostomy, rectal cancer, total mesorectal excision, Turnbull–Cutait technique

## Abstract

**Background:**

This study aimed to compare the standard one‐stage coloanal anastomosis (CAA) technique plus diverting ileostomy and the Turnbull–Cutait (T–C) technique with delayed CAA in terms of early post‐operative morbidity in patients with low rectal cancer.

**Methods:**

A total of 33 patients with non‐metastatic distal rectal cancer who were operated with one of the two different reconstruction methods (one‐stage CAA plus diverting ileostomy or two‐stage T–C technique with delayed CAA) after total mesorectal excision were included in this retrospective study. The two groups were compared for early post‐operative morbidity within 30 post‐operative days using complication frequency, Clavien–Dindo classification and Comprehensive Complication Index scores.

**Results:**

The two groups did not differ in terms of morbidity parameters, including frequency of any morbidity, presence of grade 3b morbidity requiring management under general anaesthesia, as well as Comprehensive Complication Index score (*P* > 0.05 for all).

**Conclusion:**

Our findings suggest that the two techniques did not differ in terms of early post‐operative morbidity. Owing to its comparable morbidity and safety to CAA plus concomitant ileostomy performed at the same session, the T–C technique may be considered in distal rectal cancer patients refusing to have a temporary stoma and in patients in whom CAA poses technical difficulties during the initial operation.

## Graphical abstract

This study aimed to compare the standard one‐stage coloanal anastomosis (CAA) technique plus diverting ileostomy and the Turnbull–Cutait technique with delayed CAA in terms of early post‐operative morbidity in patients with low rectal cancer, and found that the two techniques did not differ in terms of early post‐operative morbidity. Thus, the Turnbull–Cutait technique may be considered in distal rectal cancer patients refusing to have a stoma and in patients in whom CAA poses technical difficulties during the initial operation.

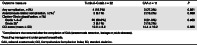

## Introduction

Although total mesorectal excision (TME) followed by coloanal anastomosis (CAA) represents a well‐established treatment modality for low rectal cancers, it still remains a challenging procedure for surgeons.^1^ Reconstruction after resection generally involves a one‐stage CAA in conjunction with protective ileostomy performed in the same session. However, subsequent anastomotic leakage may be extremely disappointing for the patients and surgeons alike. CAA without creating a protective stoma is associated with even higher rates of anastomotic leakage.^2^ Anastomotic fistulas and pelvic complications may not only undermine the function of the sphincter muscles, but also delay adjuvant treatments and increase the risk of local recurrence, and thus have potential adverse effects on oncological outcomes.^1^, ^3^, ^4^

In the early 1950s Turnbull in Cleveland Clinic and Cutait in Brazil simultaneously introduced the two‐stage transanal anastomosis technique in an attempt to reduce the morbidity associated with colorectal anastomosis.^5^, ^6^ Originally, the described technique was indicated in patients with midrectal cancer and in children with Hirschsprung's disease as a two‐stage pull‐through procedure. The first stage included the resection of the affected segment and pull through of the remaining distal colon through the anus. The second stage of the procedure was performed after several days and consisted of a delayed CAA, thus avoiding the creation of a stoma. This procedure was largely abandoned due to the introduction of stapling anastomotic devices.

In recent years, we have witnessed a resurgence of the Turnbull–Cutait (T–C) technique to save patients from a permanent stoma in complicated conditions such as persistent recto‐urethral and recto‐vaginal fistula as well as chronic pelvic sepsis due to colorectal anastomotic leak or other causes.^7^, ^8^, ^9^ Furthermore, some surgeons routinely use this technique in low rectal cancers.^10^, ^11^

This study aimed to compare the two techniques performed after TME, that is, two‐stage T–C technique with delayed CAA and classical technique of CAA with covering ileostomy, in terms of early post‐operative morbidity.

## Methods

### Patients

A total of 33 patients with non‐metastatic distal rectal cancer who were operated with one of the two different reconstruction methods after TME were included in this retrospective study. Patients suitable for both techniques were informed about the two procedures and were asked to choose one of them. The T–C procedure was used in patients who did not wish to have a stoma, even if temporary. Twenty‐two patients were operated with two‐stage T–C technique and the remaining 11 had CAA and simultaneous protective ileostomy. All operations were performed by two experienced colorectal surgeons. The two groups were compared for early post‐operative morbidity within 30 post‐operative days using complication frequency, Clavien–Dindo classification^12^ and Comprehensive Complication Index (CCI) scores.^13^, ^14^ For this purpose, early post‐operative phase was defined as the 30 days that elapsed after the second stage operation for the T–C group and after the initial operation in the CAA plus concomitant ileostomy group. The study protocol was approved by the local ethics committee of the Acibadem Mehmet Aydinlar University (date, 9 January 2020; no. ATADEK‐2020‐01/11) and the study was conducted in accordance with the ethical standards laid down in the Declaration of Helsinki and its later amendments.

### Surgical techniques

#### Two‐stage T–C technique with delayed CAA

**Fig 1:**
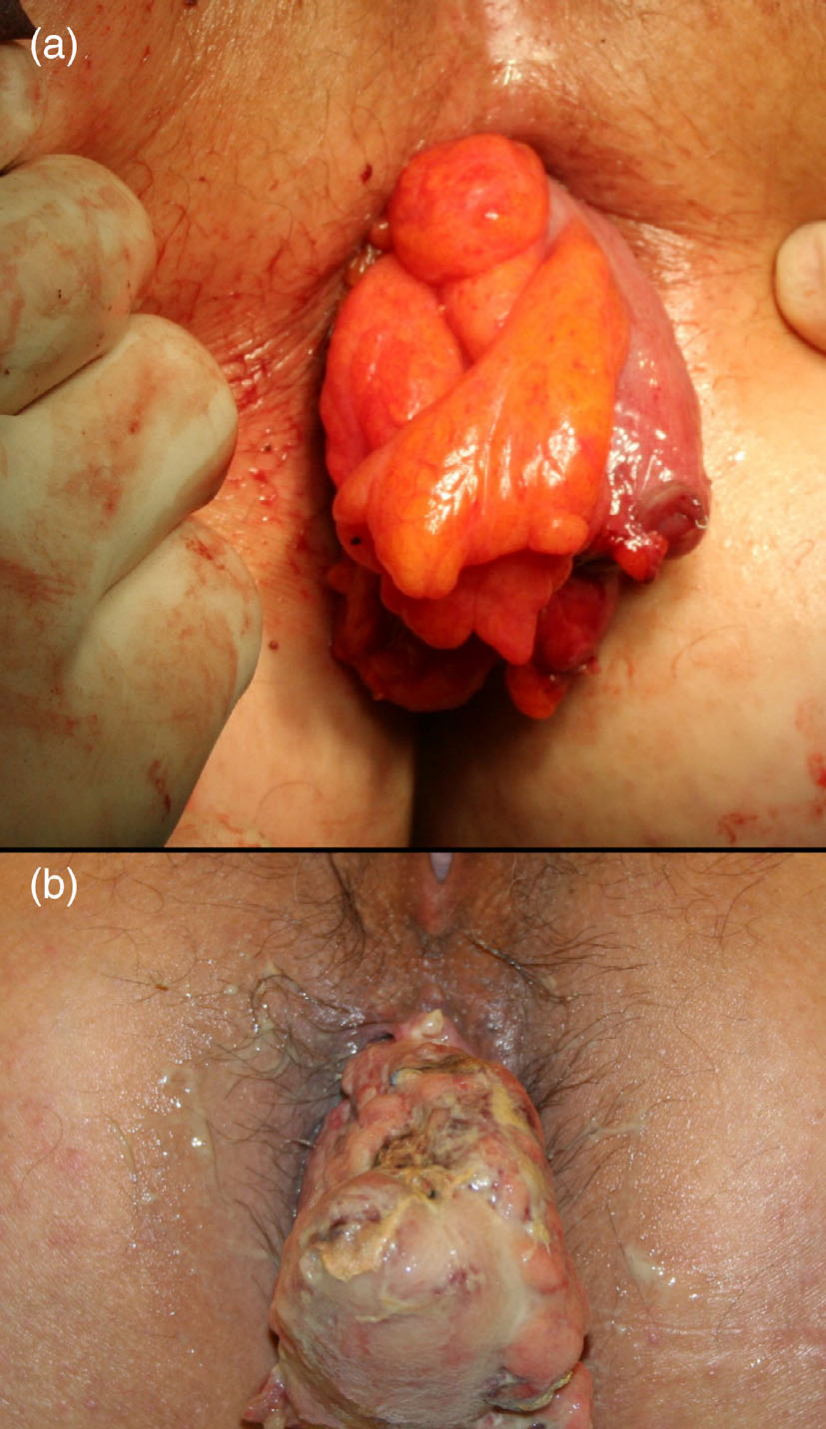
View of the exteriorized colon segment. (a) Intraoperative view of the colonic stump at the end of stage 1 and (b) post‐operative sixth day view of the colonic stump.

In the first stage, the patients were placed in the Lloyd–Davis position, and an abdominoperineal approach was used. In the abdominal phase, following an abdominal incision, conventional very low anterior resection with TME was carried out in accordance with the oncological principles of no‐touch technique, high vascular ligation and nerve sparing. After complete splenic flexure mobilization, the inferior mesenteric vein was ligated and divided close to the ligament of Treitz. The inferior mesenteric artery was isolated, ligated and divided 1 cm above the aorta. Dissection was advanced to the pelvic floor through the avascular plane (holy plane) between the parietal sheet of endopelvic fascia (presacral fascia) and fascia propria encircling the mesorectum sown to the level of the levator ani musculature. A drain was placed in the pelvis. In the perineal phase, a Lone‐Star retractor (Lone‐Star Medical Products, Stafford, TX, USA) was inserted to retract the anus. Mucosa and the internal anal sphincter muscle were circumferentially incised until reaching the intersphincteric plane using a monopolar diathermy. The incision level was made depending on the location of the low rectal cancer: a 1‐cm tumour‐free distal margin was aimed for and hence the incision was made at the level of dentate line up to 1 cm above the level of the dentate line. The cranial lumen was closed with a purse string suture and dissection was continued posteriorly until the abdominal pelvic dissection plane was reached. Before pulling the colon through the anal canal, four 3/0 polyglactic acid sutures were placed at the cardinal points of the anal canal, as high as possible, taking bites of the upper edge of the divided internal sphincter thereby avoiding full‐thickness damage to the muscle. The rectum and sigmoid colon were then pulled through the anal canal and divided proximally at the level of the ligation of the left colic artery. A colonic segment of about 8–10 cm was left outside. Finally, the colonic exteriorized segment was fixed to the perianal skin with four to six 4/0 absorbable sutures (Vicryl, 4/0; Ethicon, Cincinnati, OH, USA) and wrapped in wet gauze. Figure 1 shows the intraoperative and post‐operative appearance of the exteriorized colon segment.

**Fig 2:**
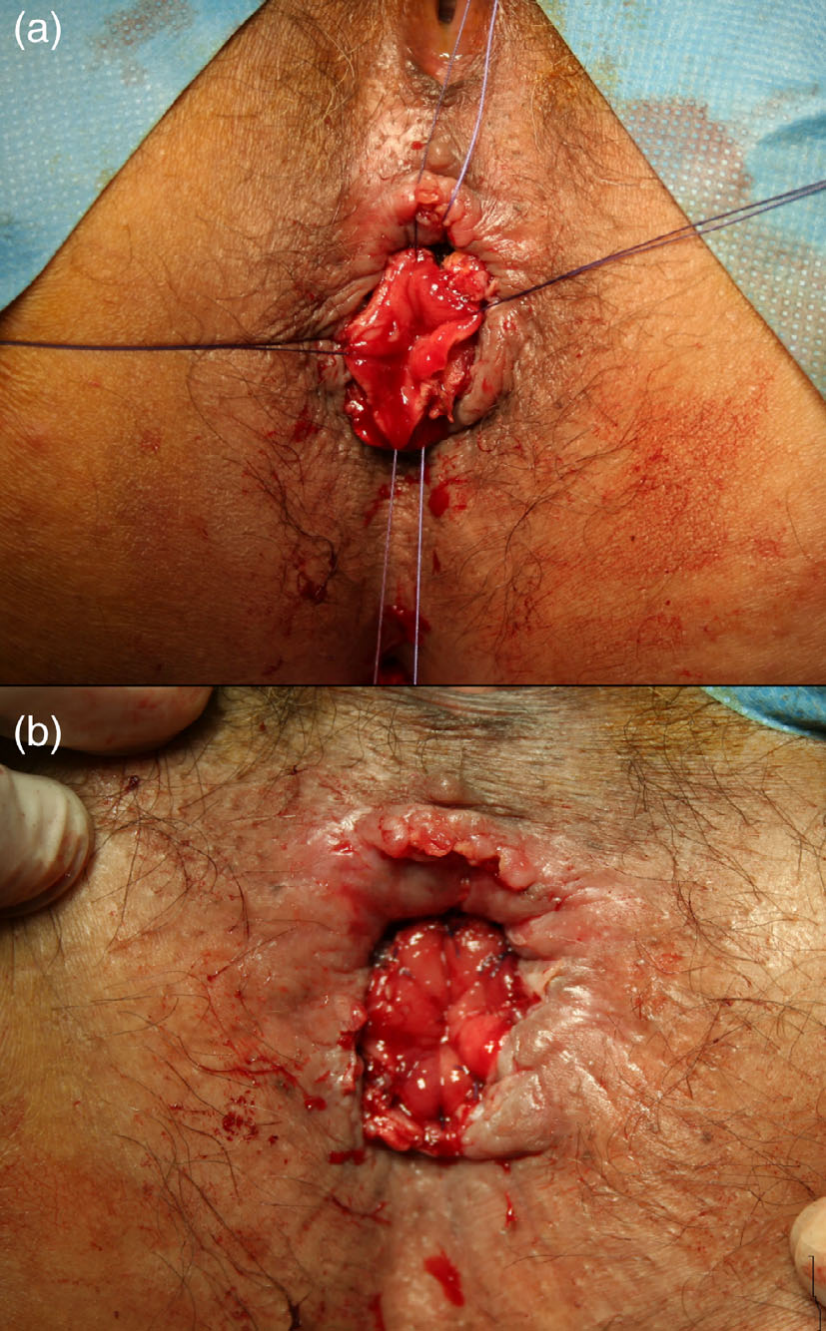
View of delayed coloanal anastomosis (CAA). (a) During CAA and (b) after the completion of CAA.

The second surgical stage of delayed CAA was performed between post‐operative days 5 and 7 under sedation and epidural anaesthesia. The patient was placed in the lithotomy position. No retractors were needed, and the adhesions between the anal canal and colon were preserved. After tying off the mesocolon at the level of the anal verge, the exteriorized segment was divided with cautery. A hand‐sewn CAA was performed using 8–12 interrupted sutures at the dentate line level. Figure 2 shows the view of delayed CAA, during and after its completion. The lumen was then checked with anoscopy.

### CAA plus concomitant ileostomy

Patient position and abdominal phase of the operation was the same as with the T–C technique. At the perianal phase, a Lone‐Star retractor was placed in the perineum and then the mucosa and internal sphincter muscle were circumferentially incised at the dentate line with monopolar diathermy until reaching the intersphincteric plane, and continued posteriorly to reach the abdominal dissection plane. Proximal colon was divided at the level of the left colic artery and the resection specimen was removed with a pull‐through technique. CAA was done using 12 interrupted hand‐sewn 4/0 Vicryl sutures, after confirming the presence of active mucosal bleeding and absence of tension on anastomosis. A loop ileostomy was created in the right lower quadrant.

### Post‐operative care and follow‐up

After CAA plus concomitant ileostomy, oral fluid intake was initiated on the first post‐operative day. After the T–C technique, oral intake was not allowed and the patient received total parenteral nutrition (2000 kcal/day) during the period between the two stages. Patients were advised to lie on their side or spread their legs when they lay in the supine position. The colonic stump viability was checked once daily. Bladder catheter was removed after the beginning of urge for urination upon clamping of the catheter. Intraoperative drain was removed when daily drainage was below 30 mL.

### Statistical analysis

For statistical analysis, SPSS version 21 (Armonk, NY, USA) for windows was used. Descriptive data were presented as mean ± standard deviation or number (frequency), where appropriate. Hypothesis tests and graphical methods were used to test normality of the data. For the comparison of continuous variables, Student's *t*‐test for independent samples or Mann–Whitney *U*‐test was used, depending on the distribution of data. Pearson's chi‐squared test or Fisher's exact test was used for the comparison of categorical data. A *P*‐value of <0.05 was considered an indication of statistical significance.

## Results

### Patient characteristics

All patients had non‐metastatic distal rectal cancer. Table 1 shows demographical and clinical characteristics of the two groups. The two groups did not differ regarding age, body mass index or distance from the anal verge. The two groups were also similar regarding gender, T status or neoadjuvant treatment distribution. However, N‐positive disease was less frequent among patients who were operated with the T–C technique (36.4% versus 72.7%, *P* = 0.049). In addition, duration of hospitalization was significantly longer in the T–C group (11.4 ± 4.2 versus 7.8 ± 2.3 days, *P* < 0.001). Among patients who underwent the T–C operation, two patients required early colostomy or ileostomy due to complications (Table 2). For the rest of this group, the mean time between the two procedures (i.e. initial operation and second stage) was 7.4 ± 1.0 days (median 7; range 6–10 days).

**Table 1:** Demographical and clinical characteristics

Characteristic	Turnbull–Cutait *n* = 22	Coloanal anastomosis *n* = 11	*P*
Age (years)	57.1 ± 10.1	52.5 ± 8.2	0.197
Male gender	11 (50.0%)	6 (54.5%)	0.805
BMI (kg/m^2^)	29.8 ± 4.0	29.0 ± 2.0	0.494
Distance from the anal verge (cm)	4.0 ± 1.5	3.3 ± 0.6	0.143
pT status^†^			
pT 0–2	10 (45.5%)	5 (45.5%)	1.000
pT 3–4	12 (54.5%)	6 (54.5%)	
pN positive^†^	8 (36.4%)	8 (72.7%)	0.049
CRM positivity	0	0	
Neoadjuvant treatment	19 (86.4%)	11 (100.0%)	0.534
Duration of hospitalization (days)	11.4 ± 4.2	7.8 ± 2.3	<0.001

Age, BMI, duration of hospitalization and distance from the anal verge data are presented as mean ± SD; other data are presented as *n* (%).

^†^ Based on pathological examination findings.

BMI, body mass index; CRM, circumferential margin; SD, standard deviation.

**Table 2:** Early post‐operative complications

Patient no.	Age, sex	Group	Day^†^	Complication	Management
1	52, M	Turnbull	2	Retraction of anastomosis	End colostomy
9	53, M	Turnbull	1	Bleeding from the exteriorized segment	Bleeding control
17	51, F	Turnbull	16	Pelvic abscess	Antibiotic treatment
19	45, M	Turnbull	2	Necrosis at the exteriorized segment	Ileostomy
23	45, M	CAA	25	Pelvic abscess	Drainage
24	67, M	CAA	2	Anastomosis leakage	Mucous fistula colostomy
30	61, F	CAA	3	Ileus	Total parenteral nutrition

^†^ Post‐operative days at which the complication developed.

CAA, coloanal anastomosis; F, female; M, male.

### Comparison of the groups for early post‐operative morbidity

An early post‐operative complication (within 30 days) developed in seven patients (21.2%). Table 2 shows the details of each complication on patient basis. On the basis of the Clavien–Dindo classification, one patient had grade 2 complications, two patients had grade 3a complications and four patients had grade 3b complications requiring management under general anaesthesia. Table 3 shows the comparison of the two groups in terms of early post‐operative morbidity outcomes. The two groups did not differ in terms of any morbidity, presence of grade 3b morbidity requiring management under general anaesthesia, as well as CCI score (*P* > 0.05 for all comparisons).

**Table 3:** Comparison of the groups for early post‐operative morbidity

Outcome measure	Turnbull–Cutait *n* = 22	CAA *n* = 11	*P*
Any complication, *n* (%)	4 (18.2%)	3 (27.3%)	0.661
Anastomosis‐related complication, *n* (%)^†^	2 (9.1%)	2 (18.2%)	0.586
Clavien–Dindo classification, *n* (%)			
Grade 1–3^†^	20 (90.9%)	9 (81.8%)	0.586
Grade 3b^‡^	2 (9.1%)	2 (18.2%)	
CCI score (mean ± SD)	12.3 ± 8.2	14.4 ± 10.2	0.665

^†^ Complications that occurred after the completion of CAA (anastomosis retraction, leakage or pelvic abscess).

^‡^ Requiring management under general anaesthesia.

CAA, coloanal anastomosis; CCI, Comprehensive Complication Index; SD, standard deviation.

No serious discomfort due to the exteriorized colon segment was observed during the period between the two procedures of the T–C technique. The friction feeling at the perineum during mobilization and concerns of a possible complication that may result from movement were the most frequently encountered patient concerns. As the distal end of the exteriorized colon segment was not open, no discharge and discharge‐related complaint was seen.

Formal assessment of function was not carried out in this study, but based on our observations, the most common complaints of patients who underwent the T–C procedure within the first three post‐operative months were incontinence for watery stool and fragmented defecation due to incomplete emptying of the colon. These functional problems were seen approximately in half of the patients and were managed satisfactorily in most cases with rectal enema application in the mornings.

### Potential factors that may affect early post‐operative morbidity

Table 4 shows comparison of the patients who had post‐operative morbidity versus patients with an uneventful post‐operative course in terms of potential factors other than surgery type. None of the parameters tested showed an association between early post‐operative morbidity (*P* > 0.05 for all comparisons).

**Table 4:** Other potential factors that may affect early post‐operative morbidity

Factors	Uneventful post‐operative course *n* = 26			Any morbidity with 30 days *n* = 7			*P*
	All (*n* = 26)	T–C (*n* = 18)	CAA (*n* = 8)	All (*n* = 7)	T–C (*n* = 4)	CAA (*n* = 3)	
Age (years)	56.1 ± 10.1	58.6 ± 10.5	50.5 ± 6.6	53.4 ± 8.1	50.3 ± 3.6	57.7 ± 11.4	0.521
Male gender	12 (46.2%)	8 (44.4%)	4 (50.0%)	5 (71.4%)	3 (75.0%)	2 (66.7%)	0.235
BMI (kg/m^2^)	29.3 ± 3.2	29.7 ± 3.7	28.3 ± 1.4	30.5 ± 4.4	30.1 ± 5.9	31.0 ± 2.1	0.472
Distance from AV (cm)	3.8 ± 1.4	4.4 ± 1.6	3.3 ± 0.7	3.6 ± 0.7	3.8 ± 1.0	3.5 ± 0.5	0.880
T group^†^							
pT 0–2	11 (42.3%)	8 (44.4%)	3 (37.5%)	4 (57.1%)	2 (50.0%)	2 (66.7%)	0.674
pT 3–4	15 (57.7%)	10 (55.6%)	5 (62.5%)	3 (42.9%)	2 (50.0%)	1 (33.3%)	
Neoadjuvant treatment	24 (92.3%)	16 (88.9%)	8 (100.0%)	6 (85.7%)	3 (75.0%)	3 (100.0%)	0.523
pN‐positive disease^†^	12 (46.2%)	7 (38.9%)	5 (62.5%)	4 (57.1%)	1 (25.0%)	3 (100.0%)	0.688

Data are presented as mean ± SD or n (%), where appropriate. *P*‐value is for the difference between patients with versus without morbidity.

^†^ Based on pathological examination findings.

AV, anal verge; BMI, body mass index; CAA, coloanal anastomosis; SD, standard deviation; T‐C, Turnbull–Cutait.

## Discussion

In this study, comparing two‐stage T–C technique and delayed CAA versus CAA performed in the initial operation with protective ileostomy in patients with distal rectal cancer, no differences were found with respect to early post‐operative surgical morbidity. To the best of our knowledge, ours is one of the few studies comparing these two surgical approaches in terms of early post‐operative morbidity exclusively in patients with low rectal cancer.

Although manual or mechanical one‐stage CAA with prophylactic ileostomy is considered the standard of care following TME for low rectal cancer,^1^ anastomotic leaks remain a major challenge associated with this procedure. In this setting, the general approach to reduce the risk of leak involves the creation of a diverting ileostomy. However, diverting ileostomy requires further reversal surgery associated with a morbidity of 17% and mortality of 0.4%, in addition to lowering the quality of life and posing the burden of stoma care for the patient.^15^, ^16^ Furthermore, anastomotic leakage has been reported to occur even in 11–15% of patients undergoing diverting ileostomy.^17^ Likewise, not all temporary stomas can be reversed, with 3–25% becoming permanent.^18^

The significance of the study by Xiong *et al*. comparing the T–C technique and CAA with protective ileostomy in patients with rectal cancer should be emphasized, as it is the first study to test these two techniques versus each other.^19^ However, in that study, it has not been clearly described whether patients received neoadjuvant chemoradiotherapy. No patients in the T–C group had anastomotic leaks and two (2.8%) had pelvic abscess. In late CAA performed after the amputation of the exteriorized segment at the second stage of the T–C technique, an anastomotic leak is likely to present with pelvic abscess if complete dehiscence of the anastomosis does not occur. Therefore, we believe that it would be more appropriate to define the pelvic abscess formation following the T–C technique as an ‘anastomosis‐related complication’. Furthermore, the incisions involving the cutaneous, subcutaneous and half‐thickness sphincters in four patients with vascular insufficiency of the exteriorized segment during the waiting period might have contributed to the absence of leakage by preventing the retraction of the pulled‐through segment. However, this may lead to subsequent functional problems. In our series, two patients (9%) had anastomosis‐related complications, including one anastomotic retraction (leakage) and one pelvic abscess.

Among the 100 patients undergoing the T–C technique for mid or distal rectal cancer, Jarry *et al*.^10^ reported a grade 3b (Clavien–Dindo classification) surgical morbidity rate of 14%, with 10 patients having pelvic septic complications (six pelvic abscesses without evident fistula, one infected pelvic haematoma, two anastomotic fistulas and one rectovaginal fistula), and 3% having anastomotic leakage. However, when pelvic septic complications are included among anastomosis‐related complications, this figure increased to 9%, similar to our observations.

Sage *et al*. observed CCI grade 3–4 morbidity during the first post‐operative month in 20% of the 87 patients undergoing the T–C technique due to low rectal cancer.^11^ The study authors explained the higher rate of septic complications based on the fact that most patients received neoadjuvant chemoradiotherapy and had very low bulky tumours. Also, necrosis in the exteriorized colonic segment was found in nine patients (10.6%). In that study, the surgical specimen was removed from the perineum following laparoscopic TME. This approach may increase the risk of injury and subsequent vascular insufficiency in the pull‐through mesenteric colon segment, particularly in patients with bulky tumours and male patients with high body mass index.^1^ In our patient group, low rate of necrosis (i.e. 4.5%) in the exteriorized colonic segment may be accounted for using an open surgical technique.

In one 2019 review by Portale *et al*. involving eight studies where patients underwent the T–C technique for rectal cancer, the reported rates of anastomotic leak and pelvic abscess ranged between 0–10.6% and 0–25%, respectively.^20^ In the current study, the observed rates of anastomotic leakage (4.5%) and pelvic abscess (4.5%) were in line with previous reports. In studies analysing patients undergoing CAA with protective ileostomy due to rectal cancer, the reported figures for anastomotic leakage and pelvic abscess ranged between 3–20% and 3–10.5%, respectively.^21^, ^22^, ^23^, ^24^, ^25^ Among our patients, the rates of anastomotic leak and pelvic abscess (9% for both) were again consistent with the published data.

A study examining the long‐term oncological and functional outcomes of 13 middle and distal rectal cancer patients operated with the T–C technique following neoadjuvant chemoradiotherapy reported the 5‐year survival rate of 85% and no local recurrence during a mean follow‐up of 101.2 months.^26^ Regarding functional outcomes, three patients required permanent stoma (two patients due to severe rectal evacuation problem and one patient due to anastomotic stenosis), and 90% of patients had satisfactory gas and faecal continence at the end of 2 years.^26^

The T–C technique has also been used as a salvage procedure in several studies with encouraging results; thus, it may have a place in the management of such difficult clinical cases as well.^7^, ^8^, ^27^

Theoretical explanations for low rates of anastomotic leakage and pelvic septic complications in patients undergoing the T–C technique include the initially insignificant effect of the retraction on the pulled‐through colonic segment due to contracted pelvic diaphragm at the termination of surgery as there is no anastomosis as well as by the prevention of the retraction of the colonic segments due to adhesions forming between the exteriorized colonic segment and anus during the waiting period.^10^ Surgeons should consider the expectations and preferences of patients with rectal cancer and aim at achieving optimum therapeutic results with minimum morbidity without compromising the oncological outcomes. Major factors that have an impact on oncological outcomes include the complete TME with circumferential–distal negative surgical margins and the use of neoadjuvant chemoradiotherapy. The T–C technique offers a surgical option that may fulfil oncological standards without the need for a protective stoma.

Retrospective design not allowing proper case selection and small sample size that may be a source of low statistical power are the two main limitations of the study. Therefore, comparisons of the two groups may not represent ideal conditions and should be interpreted within this context. Another limitation is the lack of long‐term follow‐up for functional results. However, assessment of early post‐operative morbidity seems to be more relevant and crucial in our study as protective ileostomy was not created in our patients who underwent the T–C technique. We believe that our study also has certain strengths as it exclusively included patients with distal rectal cancer in both groups and a high proportion of patients (90%) received neoadjuvant chemoradiotherapy, providing a more homogeneous sample.

In conclusion, no significant differences appear to exist between the two techniques (i.e. T–C versus CAA plus protective ileostomy) in terms of early post‐operative morbidity. Although stoma therapy units and staff are accessible by most patients in Turkey, we believe that a stoma is a cause of significant burden and cost. Owing to its comparable morbidity and safety to CAA plus concomitant ileostomy performed at the same session, the T–C technique may be considered by colorectal surgeons in selected distal rectal cancer patients refusing to have a stoma even though it is transient as well as in patients in whom CAA poses technical difficulties during the initial operation. Furthermore, as the technique negates the need to create a protective stoma, it may avoid complications from its closure in most cases.

## Conflicts of interest

None declared.

## Author Contributions

**Osman Guner:** Conceptualization; data curation; formal analysis; investigation; methodology; project administration; supervision; writing‐original draft; writing‐review and editing. **Latif Tumay:** Conceptualization; data curation; methodology; validation; writing‐review and editing.
